# How exaptation mediates the effects of customer participation on low-cost innovation: The role of strategic flexibility

**DOI:** 10.3389/fpsyg.2022.1016524

**Published:** 2022-11-23

**Authors:** Chaoyong Tang, Yongzhi Shi, Xuechun Zhu, Yibo Li

**Affiliations:** ^1^School of Economics and Management, Agricultural University of Hebei, Baoding, China; ^2^School of Management, Jiangsu University, Zhenjiang, Jiangsu, China

**Keywords:** customer participation, exaptation, low-cost innovation, coordinate flexibility, resource flexibility

## Abstract

With the advent of emerging markets, the need for low-cost innovation to meet the rising demands of people at the base of the pyramid has increased significantly. Although the critical influence of customer participation on new product development has been recognized, there have been few studies on the effects of customer participation on low-cost innovation. This study builds a moderated mediation model and explores the roles of customer participation on low-cost innovation. Based on the exaptation and strategic flexibility theories, the mediating role of exaptation and the moderating role of strategic flexibility are emphasized. A survey of 348 firms revealed that customer participation positively impacted both exaptation and low-cost innovation. In addition, exaptation mediated the correlation between customer participation and low-cost innovation. Resource flexibility negatively moderated the correlation between customer participation and exaptation and negatively moderated the mediating effect of exaptation. Furthermore, coordinate flexibility positively moderated the correlation between customer participation and exaptation and positively moderated the mediating effect of exaptation.

## Introduction

Innovation warrants the continuous investment of resources. Even companies in the USA and Europe have a product innovation success rate of only 5–10%. Thus, many of the world’s top 500 enterprises, such as GM and Panasonic, have altered their innovation models to fulfill users’ needs at the bottom of the pyramid in emerging markets through low-cost innovation (Zhu and Chen, 2014). Hence, realizing low-cost innovation characterized by shorter time, lower cost, and lower risk has become a crucial strategic choice to address the problems of insufficient supply, weak consumption capacity, and the pursuit of cost-effective products and services in emerging markets. The role and value of customer participation in innovation have been increasing recognized, seen in the proposal of consumption-centered innovation theory and the transformation of the innovation paradigm of a single subject centered on the enterprise to open innovation of multiple subjects, especially the transformation from product-led logic to service-led logic. The establishment of offline experience stores by Apple, Huawei, Xiaomi, and other companies and the actions of Dell, P&G, Haier, amongst others, to build interactive virtual communities reflect the significant contribution of customer participation to enterprise innovation. Customer participation can provide innovation sources, demand preference, and correction suggestions for market-oriented product innovation (Zhang and Liao, 2020), weakening information asymmetry and transaction costs, and saving innovation costs. Conversely, customer participation can be embedded in product design, R&D, manufacturing, and marketing (Lin and Huang, 2013; Liu et al., 2020), improving product market matching and decreasing the risk of innovation failure. Accordingly, customer participation could be a feasible path for low-cost innovation. However, there is little comprehensive discussion on the topic in the literature.

A comprehensive analysis of the correlation between customer participation and low-cost innovation requires an exploration of the mediating mechanism and situational conditions of the correlation between them. Exaptation is a concept from evolutionary biology that can be applied as a reverse innovation mechanism for the creation of markets from products and finding answers before looking for problems. However, although this paradigm of innovation through the transfer of product functions often occurs in the process of product innovation, it has received little attention (Ren et al., 2019). Per Dew et al., (2004), Ganzaroli and Pilotti, (2011), Andriani et al., (2017), specifically, the roles of customer innovation and consumer feedback as positive driving forces for exaptation. Customers not only contribute relevant market information but also provide an extended forum for the interaction between customers and enterprises. Exaptation closely correlates with customer participation. In addition, exaptation contributes significantly to innovation, which is more likely to create low-cost innovation, even producing significant changes or opening up a new market (Ren et al., 2019). Thus, investigating the mechanisms through which customer participation drives low-cost innovation from the viewpoint of exaptation will help to elucidate the “black box” of the relationship between customer participation and low-cost innovation. In contrast, the information and knowledge obtained through customer participation, such as demand preference, cognition, evaluation, and innovative ideas, should be effectively integrated into the exaptation process to indirectly promote low-cost innovation, allowing enterprises to survive and grow in a complex, changeable, uncertain, and unpredictable environment. In this case, the identification, evaluation, absorption, and application of customer information, as well as the ability of enterprises to reconstruct resources, capabilities, business processes, and strategies, determine the circumstances in which customer participation can exert a significant indirect impact on low-cost innovation (Zhang and Chen, 2014; Li and Gao, 2017). In a transition economy, the strategic flexibility of an organization, balancing internal and external needs, responding to environmental changes and allowing the flexible allocation and reconfiguration of organizational resources, processes, and strategies (Sanchez 1995; Hu and Yu 2017), allows the incorporation of information and ideas derived from customer participation into exaptation, thereby promoting internal product R&D, technology, and knowledge innovation. Hence, research on customer participation-driven low-cost innovation and strategic flexibility will elucidate the mediating mechanism of exaptation and determine the moderation mechanism of customer participation affecting low-cost innovation.

The academic value of this study is primarily reflected in the following: (i) the study discusses the creation of low-cost innovation from a new perspective. Current research has largely focused on investigating low-cost innovation in terms of intellectual capital, innovation networks, and resource bricolage, with a limited investigation of the effects of customer participation. In addition, this study demonstrates a positive correlation between customer participation and low-cost innovation, thus contributing to existing knowledge and future research into low-cost innovation. (ii) Based on the exaptation theory, this study examines the mediating role of exaptation between customer participation and low-cost innovation. The previous literature has not explored the mechanism mediating the relationship between customer participation and low-cost innovation. Thus, this study introduces the exaptation variable, demonstrating that customer participation could improve low-cost innovation through exaptation, further enriching the new idea of the correlation between customer participation and low-cost innovation. (iii) This study integrates strategic flexibility into the theoretical model of customer participation and low-cost innovation, discusses the moderating role of strategic flexibility, and explores the boundary conditions of customer participation affecting low-cost innovation.

## Theoretical background

Low-cost innovation describes the use of various methods by enterprises to realize innovation in a low-cost manner and decrease costs through innovation (Tang et al., 2022). Low-cost innovation is realized through specialization, imitation learning, R&D, design innovation, technological innovation, and diffusion (Chen and Ren, 2011). Other studies have highlighted that low-cost innovation embodies comprehensive innovation efficiency based on transforming and integrating traditional management elements, such as technology, design, market, and organization. Its essence is to fulfill the value needs of customers faster and more effectively. Moreover, it is an innovative model with comparative advantages within the homogeneous industrial market. In addition, low-cost innovation is not a low-resource investment in an absolute sense but an innovation process characterized by low financial and time costs, as well as a low market risk compared with competitors (Cai et al., 2014). Several previous studies have investigated mechanisms associated with low-cost innovation from different angles. At the qualitative research level, Hou and Zhang (2013) examined the mechanism of low-cost innovation, including the use of embedded networks, resource absorption, and emergent innovation, from the viewpoint of a resource view. Chen and Chen (2013) analyzed the formation of low-cost innovation from the strategic level, including the demands of enterprise innovation practice and the theory of endogenous comparative advantage to support the evolution of low-cost innovation. Suneel (2018) claimed that technology diffusion–oriented policies and weak industry-university linkages play a vital role in low-cost innovation from the standpoint of the industry-university research. At the empirical research level, Zhu and Chen (2014), Zha et al., (2015), Shi and Su (2018), and Tang et al., (2022) investigated the creation of low-cost innovation from the viewpoints of innovation networks, learning from failures, knowledge management, intellectual capital, and resource bricolage. Clearly, the realization of low-cost innovation has become a significant issue in academic circles.

Academics have interpreted the concept of customer participation from different viewpoints. From the standpoint of resources, some studies proposed that customer participation is realized by investing different resources into the products or services (Kelley et al., 1990). From the behavior perspective, several studies also claimed that customer participation denotes a type of behavior involvement of customers in the process of product design, development, production, and delivery (Chi et al., 2012), which exists in the continuous interaction between employees and customers (Li and Hsu 2018). From the value creation perspective, other studies defined customer participation as any form of customer participation in service value creation (Yi et al., 2011). In comparison, the definitions of Yao and Wang (2012) are more comprehensive and specific, defining customer participation in terms of enterprise involvement of customers in various ways during the development of new products. Customers not only provide new ideas about products but can also design and develop new products in association with enterprises and can even take the lead in testing and using new products. Current research has demonstrated the importance of customer participation in product innovation, new product development, and dual innovation through information exchange, and this participation in the creation and collaborative cooperation has generally been recognized by the academic community (Yao and Wang 2011; Cees and Fardad 2014; Liu et al., 2020; Zhang and Cai 2020; Sun et al., 2022; Tian et al., 2022). A close correlation exists between customer participation and enterprise innovation; however, the existing literature lacks research on the correlation between customer participation and low-cost innovation. In situations of economic transformation, an understanding of the ways in which customer participation impacts and influences the path of low-cost innovation is key.

Schumpeter claimed that innovation involves new products, new processes, and the application of existing technologies in new fields (Li 2005). Existing research has systematically discussed new products and processes; however, the application of the existing technologies in new fields, that is, the phenomenon of exaptation, has received little attention (Ren et al., 2019). The concept of exaptation originates from evolutionary biology, and Mokyr (2000) first introduced exaptation into management, interpreting it as “the unexpected success of a technology used in another field.” Subsequently, Gregory (2008) considered exaptation to denote existing components, which initially had no or additional functions, rearranged and combined into a new, more complex form, giving them new functions. Arthur (2007) explained exaptation as when a new environment appears, or new requirements appear in an application field, the old technology or old basic principles are used to adapt to the new environment through “extension.” In addition, Ganzaroli and Pilotti (2011) described exaptation in terms of the exploitation of the availability of existing functions to perform a new function or select an environment to stimulate their potential. Drawing on these researchers’ viewpoints, this study holds that exaptation denotes the process that existing products and technologies have new functions through the application of new fields and new environments, or through integration and reconstruction. Existing studies have demonstrated that as an innovation method with situational characteristics, exaptation plays a key driving role in enterprise technological innovation, produces major changes in a specific field, creates a new technology track, or opens up a new industry, and can provide innovation power and support for the long-term sustainable development of enterprises (Ren et al., 2019). Undeniably, exaptation is an unexpected result, with some luck factors; however, it can also increase the probability of exaptation through the influence of enterprise level (Ren et al., 2019; Li et al., 2021). For example, enterprises can stimulate exaptation through three links—expansion pool, expansion activities, and expansion forum (Garud et al., 2018)—and improve the likelihood of exaptation by accumulating diversified knowledge and enhancing the ability to seize opportunities (Dew et al., 2004; Andriani et al., 2017). Other studies demonstrated that purposeful research and coincidence triggered exaptation and discussed the feasibility of applying exaptation to drug emergency research and development (Li et al., 2022). Customer innovation is a critical factor in the antecedent research on exaptation. Research has shown that customers may discover many paths to exaptation, and customer participation closely correlates with exaptation because customers can modify existing products based on their experience, psychological framework, educational background, and other subjective judgments, or use them in a way with novel functions, resulting in exaptation (Andriani et al., 2017). Ganzaroli and Pilotti (2011) highlighted that linking the existing knowledge system with customer needs and realizing the two-way interaction between producers and customers is helpful to realize exaptation. The research logic of exaptation-driven innovation from the standpoint of customer participation remains unclear, and there is a lack of empirical research, which still warrants further investigation.

Katsuhiko and Hitt (2004) claimed that strategic flexibility denotes the ability of enterprises to identify changing factors in the environment and quickly invest resources in the new environment. Sanchez (1995) further divided strategic flexibility into resource flexibility and coordination flexibility. Resource flexibility denotes the flexibility of resources owned by enterprises, which is reflected in the multi-purpose, shareability, and convertibility of enterprise resources. Coordination flexibility is reflected in organizations’ ability to share, transform, and network resources. Existing studies have reported that strategic flexibility not only exerts a positive predictive impact on product innovation (Yang et al., 2016; Feng and Liu 2021), strategic innovation, management innovation (Han and Gao 2017), strategic change (Chen et al., 2018), and competitive advantage (Xu 2021) but also a certain moderating effect on innovation and other related outcome variables. Ye et al., (2015) reported that strategic flexibility could moderate the correlation between knowledge heterogeneity and enterprise innovation performance. Li and Gao (2017) explored the moderating effect of strategic flexibility on the correlation between knowledge-sharing and cooperative original innovation. Li et al., (2018) pointed out that strategic flexibility increases the impact of environmental scanning on dual innovation. This study introduces strategic flexibility into the theoretical model and discusses the moderation mechanism of customer participation driving low-cost innovation from the viewpoint of resource flexibility and coordination flexibility.

Although the studies mentioned above have obtained good theoretical results, some problems still warrant further discussion. (i) Most studies have fully explored the role of customer participation in driving product innovation, technological innovation, and other outcome variables. Low-cost innovation, specifically, as a crucial form of innovation, merits further investigation in terms of its relationship with customer participation. (ii) Previous studies have investigated the mechanism linking customer participation, product development, and dual innovation from the viewpoints of information-sharing (Yao and Wang 2011), correlation-embedding (Yao and Wang 2012), organizational learning (Yao et al., 2015), and pressure (Liu et al., 2020). Introducing exaptation into the theoretical framework of the correlation between customer participation and low-cost innovation and elucidating the way in which exaptation mediates the relationship between them is worth investigating. (iii) Previous studies have paid little attention to moderation of the correlation between customer participation and low-cost innovation. This study analyzes both the nature of this relationship and changes in its strength in terms of strategic flexibility, which would help to make up for the lack of research on the moderating mechanism between customer participation and low-cost innovation. Accordingly, to make up for limitations in current research, this study introduces two variables, namely, strategic flexibility and exaptation, and constructs a moderated mediation model, together with an examination of the mechanism underlying the impact of customer participation on low-cost innovation. First, the study discusses the impact of customer participation on low-cost innovation. Second, it analyzes the mediating role of exaptation between customer participation and low-cost innovation. Third, strategic flexibility is introduced as a moderator of the correlation between customer participation and low-cost innovation. This study not only investigates the internal logic and situational mechanism of customer participation affecting low-cost innovation but also provides new insights into China’s manufacturing enterprises to implement the path of low-cost innovation.

## Hypotheses

### Customer participation and low-cost innovation

As a crucial knowledge source of technological innovation, customer participation can be understood as the degree to which customers are involved in activities involved in the development of new products, reflecting customers’ innovative wisdom, creativity, and innovative value (Fang 2008). It can not only provide information on consumer preferences, market demands, and development opportunities, decrease “information stickiness,” reduce market mismatch risk, and enhance the likelihood of successful innovation (Zhang and Liao 2020) but can also establish a means for collaborative innovation and development between enterprises, which is helpful to promote market-oriented technological innovation as well as improving innovation and the development of new products.

Customer participation can not only contribute novel and creative ideas to product innovation but can also lead to the development of new products with low financial and time investments and low market risk (Lin and Huang 2013). Füller et al., (2006) highlighted that customers could provide enterprises with high-quality problem solutions or innovative ideas and creative suggestions, markedly decreasing the market risk of innovation. Tao et al., (2019) and Zhang and Cai (2020) showed that customers could become involved in the product development process by generating creative ideas, designs, and development, as well as through product testing, both online and offline, thus reducing the cost of information acquisition and transfer through rapid and efficient information exchange, transmission, and sharing, which has the characteristics of “learning by doing” and “trial and error,” to improve the success rate of innovation and decrease the cost of innovation and time to market. Zhang and Liao (2020) claimed that customers participating in R&D provide enterprises with demand, design, evaluation, and other information, which decreases the information asymmetry between enterprises and customers. Meanwhile, enterprises and customers are integrated into the product innovation process, which can develop new products at low cost in the shortest time and shorten the commercial application cycle of new products. Accordingly, we hypothesize that:

*H1*: Customer participation exerts a significant positive impact on low-cost innovation.

### Mediation effect of exaptation

As a crucial source of innovation, exaptation involves not only the interaction of elements, such as the accretion of diversified knowledge, construction of innovative culture, and organizational structure convenient for open innovation but is also an innovation model requiring customer participation. Cees and Fardad (2014) highlighted that enterprises not only absorb, integrate, and apply their diversified knowledge and give play to their creativity through customer participation in the innovation process but also promote the commercialization process of innovation activities faster and more economically. This study claims that the impact of customer participation on exaptation can be summarized into two aspects: ① the role of information supply. Customers can modify existing products through their subjective judgment or use them in novel ways to fulfill specific needs. When these needs emerge from the mixture of personal experience, psychological framework, education, and other activities, they create a new environment, which leads to exaptation (Andriani et al., 2017). In the 1990s, based on customer feedback, Haier rapidly launched washing machines with functions such as washing sweet potatoes, fruits, and clams, and there are cases in the software market where users modified existing software to solve specific problems, illustrating the mechanism through which customers proposed new ideas and additional requirements through product prototype experience to promote exaptation. It is necessary to connect the existing knowledge system with possible new fields and new needs (Ganzaroli and Pilotti 2011); that is, customer participation can activate this specific knowledge and technologies through its unique ability to transfer knowledge, increasing the likelihood of new applications of existing products or technologies (Tian et al., 2022). ② The significance of value creation. Huikkola et al., (2013) showed that when customers cooperate with enterprises in R&D, they tend to be involved in the lifecycle of innovation activities, contributing human capital, enhancing subjective initiative and creativity, and performing organizational learning and knowledge-sharing activities to enhance novel applications of existing products and technologies. In addition, the discovery of new uses for the product and changing its functional modules can lead to exaptation. For example, laughing gas was initially used for entertainment and was later used in the medical field, an example of customer participation in innovation (Ren et al., 2019). As shown by Yao et al., (2015), customer participation in innovation leads to more frequent interaction between personalized customer needs and enterprise technology, enabling expansion and a knowledge reserve, and possible application opportunities for exaptation through situational mechanisms (Andriani et al., 2017). Accordingly, we hypothesize that:

*H2*: Customer participation exerts a significant positive impact on exaptation.

Exaptation has the advantages of faster speed, lower cost, and less risk, promoting low-cost innovation, and even opening a new technology track at low cost (Ren et al., 2019; Li et al., 2021). First, the development of existing resources (e.g., technologies and products) can save financial costs. Exaptation demonstrates that can use existing products and technologies for low-cost innovation (Mitsuru and Tomoatsu 2014). Exaptation can decrease the cost of new product development and new technology acquisition. In contrast, unlike new products, new processes, and other innovations, exaptation allows the functional transfer of existing products or the successful application of existing technologies in new fields. Mining or extracting the potential value of existing technology is often much cheaper than developing new products for a new function. Second, as a reverse innovation method (scheme precedes problem), exaptation is a crucial strategic tool for the execution of low-cost innovation. Enterprises can dynamically couple the current reserves of knowledge and technology with external environmental opportunities through exaptation, actively adapt to the market environment, and match new market opportunities, decreasing the market risk of product innovation. In addition, Andriani et al., (2017) explored exaptation in the pharmaceutical industry, reporting that each drug has 2.2 new functions, one of which is also highly innovative. In other words, each product contains potential options that can be transformed into new markets. The cultivation of shadow options expands the product’s versatility (Cattani 2006; Li et al., 2021), decreases the R&D risk, and provides the possibility and feasibility for low-cost innovation. Accordingly, we hypothesize that:

*H3*: Exaptation exerts a significant positive impact on low-cost innovation.

According to Hypotheses 2 and 3, this study believes that customer participation can act on low-cost innovation through exaptation; that is, exaptation mediates the correlation between customer participation and low-cost innovation. Specifically, as a key innovation model, exaptation effectively links the correlation between customer participation and low-cost innovation. Frequent interactions between enterprises and customers can develop extensive, unique, and heterogeneous knowledge resources, augment the knowledge base of enterprises, and help to combine the existing knowledge system with possible new fields, as well as the prediction of new environments where adaptation is possible (Ren et al., 2019). Moreover, customer information can help enterprises identify market demand and market opportunities (Sun et al., 2022), thereby helping to apply scientific discoveries in novel ways and new environments. In contrast, exaptation is often more economical than deliberately developing a new product by tapping the potential value of an enterprise’s product or technology, that is, it saves the innovation cost between customers and enterprises, curtails the process of customers participating in low-cost innovation, and then increases the speed of product innovation (Zhang and Liao 2020). In addition, as Cattani (2006) highlighted, exaptation provides a new tool for enterprise innovation, namely, the “shadow option.” When the accrued knowledge of the enterprise and the appropriate situation induce the transfer of function, the shadow option may develop into the actual value of the enterprise. In terms of the “shadow option,” building a mixed organization of customers and enterprises can identify shadow options more quickly, promote the two-way interaction between enterprises and customers, have extra space to develop unexpected new models, and decrease R&D risks, thereby providing the possibility for low-cost innovation. As described by Peng et al., (2011), to perform product innovation in the fields of high-end CNC machine tools and all-motor CNC systems, the Haitian Group specifically utilized customer information and knowledge on market demands and development trends, revitalizing some of the company’s existing technical assets (expansion adaptation), and developed market-leading technical service solutions (low-cost innovation) with low time and financial costs. Hence, customer participation indirectly affects low-cost innovation *via* exaptation. Accordingly, we hypothesize that:

*H4*: Exaptation mediates between customer participation and low-cost innovation.

### The moderating effect of strategic flexibility

The realization of customer participation-driven exaptation requires mechanisms to stimulate function transfer to promote low-cost innovation. Strategic flexibility is a situational variable connecting inside and outside and denotes the ability of enterprises to quickly identify crucial changes in the external environment and respond, adjust strategic decisions, and put resources into new schemes (Sanchez 1995; Li and Gao 2017). The concept included both resource and coordination flexibility. Resource flexibility aims to expand the application scope of resources and tap their potential uses to increase the choice of enterprises when the environment changes. Coordination flexibility reflects a firm’s ability to use resources effectively, emphasizing its ability to define, build, and integrate existing resources to support enterprise strategy.

Although resource flexibility has the advantage of enhancing the diversified use of existing resources to enhance the ability to cope with the environment, the view of “resource is commitment,” as proposed by Kraatz and Zajac (2001), often produces the result of strategic “locking,” which makes it challenging for enterprises to identify distant opportunities for exaptation from customer participation in innovation (Ren et al., 2019). The “capability trap” created by resource flexibility makes enterprises more inclined to stick to existing resources, hinders the acquisition, learning, absorption, and utilization of new knowledge, such as external market demand, and weakens the process of customer participation-driven exaptation. In addition, when enterprises are faced with the challenge of using only their own products and technologies for exaptation, further developmental opportunities should be found within both developmental trends in the industry and changes in market demand (Zhang and Liao 2020).

Coordination flexibility applies resources to new fields by identifying the direction of resource allocation, followed by reconstruction and determination of the use of resources (Hu and Yu 2017) to promote the realization of customer participation-driven exaptation. Coordination flexibility focuses on the compound innovation of existing knowledge, products, and technologies, allowing the acquisition of distant development opportunities through customer participation, enhancing the ability to tap the diversified uses of existing products and technologies, and expanding new resource applications (Li et al., 2021), and thus promoting exaptation. In contrast, coordination flexibility augments the organizational learning and absorption ability, promotes enterprises to better understand and absorb the demand information from front-end customers, and through the integrated application of existing resources and customer knowledge resources (Jiang et al., 2022), promotes the interactive coupling among existing products, technologies, and new needs, identifies and seizes opportunities to support the realization of exaptation, to increase the probability of successful implementation of exaptation. Hence, we hypothesize the following:

*H5a*: Resource flexibility negatively moderates the correlation between customer participation and exaptation.

*H5b*: Coordination flexibility positively moderates the correlation between customer participation and exaptation.

Based on the previous hypothesis, strategic flexibility (resource flexibility and coordination flexibility) moderates the correlation between customer participation and exaptation, and exaptation plays a mediating role between customer participation and low-cost innovation. This study further deduces that strategic flexibility (resource flexibility and coordination flexibility) moderates the mediating role of exaptation, creating a moderated mediation model. Specifically, customer participation can drive low-cost innovation through exaptation; however, the indirect effect of customer participation on low-cost innovation closely correlates with strategic flexibility (resource flexibility and coordination flexibility). Under high resource flexibility, the firm’s fixed thinking and organizational inertia play a leading role in innovation. Resource flexibility weakens the probability that customers’ new demands and opportunities merge with the firm’s internal knowledge to produce the function transfer of products or technologies, thereby inhibiting the indirect effects of customer participation and low-cost innovation. Under high coordination flexibility, the ability of the enterprise to allocate and reconfigure resources plays a key role in innovation. Coordination flexibility enhances the development opportunities for customers and promotes the likelihood of the application of existing products in new fields, thereby strengthening the indirect correlation between customer participation and low-cost innovation. That is, coordination flexibility increases the indirect effect of customer participation transmitted to low-cost innovation through exaptation, and resource flexibility weakens the indirect effect of customer participation transmitted to low-cost innovation through exaptation. Thus, we hypothesize the following:

*H6a*: Resource flexibility negatively moderates the mediating role of exaptation between customer participation and low-cost innovation.

*H6b*: Coordination flexibility positively moderates the mediating role of exaptation between customer participation and low-cost innovation.

**Figure 1:**
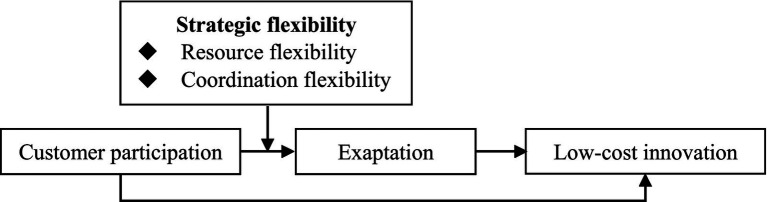
Theoretical model.

Figure 1 shows the theoretical framework.

## Data and methods

### Sample data collection

A questionnaire survey method was used to obtain the sample data, and the hypotheses were tested by investigating the managers and technicians engaged in product innovation and industry-university research cooperation in China’s manufacturing enterprises. A questionnaire survey was conducted on Shanxi, Jiangsu, Shanghai, Henan, and Hebei manufacturing enterprises. We obtained sample data on customer participation, exaptation, strategic flexibility, and product innovation. Specifically, a total of 450 survey questionnaires were distributed, of which 374 sample data were collected, and 26 invalid questionnaires containing incomplete answers and logical errors, were excluded. Finally, 348 valid questionnaires were obtained. Our sample comprised the industry including textile and clothing (18.10%), food processing (28.16%), pharmaceutical manufacturing (34.77%), and others (18.96%); the age of the enterprise was 5–15 years (14.37%), 16–30 years (70.40%), >30 years (15.23%); the nature of ownership included state-owned enterprises (51.72%), private enterprises (21.26%), foreign-funded enterprises (3.45%), collective enterprises (7.76%), and other enterprises (15.80%); the scale of enterprises included ≤300 people (3.16%), 300–1,000 people (10.63%), 1,000–2,000 people (19.83%), 2,000–3,000 people (53.74%), and >3,000 people (12.64%).

### Measures

#### Customer participation

We drew on the research design of Fang (2008), Yao et al., (2015), and Zhang and Cai (2020), and modified the scale in combination with the situation in this study. Finally, the scale was measured with six items: “customer participation or involvement is very important to research and development activities.”

#### Exaptation

Exaptation was measured with a three-item scale developed by Tang et al., (2022), such as “A technology of the enterprise has been successfully applied in another field.”

#### Strategic flexibility

We drew on the research design described by Sanchez (1995) combined with features unique to this study to modify the measurement items, and finally determined two dimensions, namely, resource flexibility and coordination flexibility. Resource flexibility had three measurement items, while coordination flexibility had four measurement items. For example, “enterprises can effectively deal with resource usage issues in a dynamic environment.”

#### Low-cost innovation

Low-cost innovation was measured with a four-item scale developed by Shi and Su (2018). For example, “enterprises develop new products and services in a low-cost manner.”

The previous literature has shown that enterprise characteristic variables could influence product innovation, including enterprise age, enterprise size, enterprise nature, and industry distribution (Xu et al., 2016; Hu and Yu 2017). Thus, these variables were controlled in this study.

## Results

### Reliability, validity, and common method bias

Using Cronbach *α* to test the reliability, we observed that the Cronbach *α* coefficients of customer participation, exaptation, resource flexibility, coordination flexibility, and low-cost innovation were 0.823, 0.758, 0.678, 0.719, and 0.741, respectively, suggesting that the scale had good reliability (Table 1). In addition, the standardized factor loading coefficients of all latent variables were >0.5, and the square root of the average variance extraction (AVE) value of all variables was more significant than the correlation coefficient with other variables (Table 2), suggesting that the research scale had a good level of validity. Meanwhile, using AVE as a convergence validity test tool, the AVE values of customer participation, exaptation, resource flexibility, coordination flexibility, and low-cost innovation were 0.648, 0.750, 0.608, 0.610, and 0.507, respectively, all >50%, suggesting that the main variables had good convergence validity. Moreover, we used the Harman method to test the homologous deviation, showing that the first factor explained 34.155% of the variation without rotation, which was <50% of the statistical requirements, indicating no significant common method deviation in the data.

**Table 1:** Measure reliability and convergent validity.

Measures	OL	CA	AVE
**Customer Participation**		0.823	0.534
Customers often provide information about needs and preferences	0.795		
The participation or involvement of customers is very important for R&D activities	0.754		
It is an important part of the product innovation process	0.753		
Customers’ creative efforts play an important role in product innovation	0.700		
Customers often put forward R&D plans	0.695		
Customers often conduct independent research and development	0.682		
**Exaptation**		0.720	0.609
A technology of the enterprises has been successfully applied in another field	0.837		
A product of the enterprises has gained new functions in the new environment	0.831		
Enterprises have new functions through the combination of existing product functions	0.743		
**Resource Flexibility**		0.678	0.609
Enterprises produce different products and services from the same resource in a wide range	0.812		
The conversion cost and difficulty of producing different products and services from the same resource are small	0.793		
The conversion time for enterprises to produce different products and services from the same resource is short	0.733		
**Coordination Flexibility**		0.719	0.543
Enterprises can find future opportunities and react faster than existing and potential competitors	0.779		
Enterprises can find new resources or their combinations faster than existing and potential competitors	0.745		
Enterprises can explore new markets faster than existing and potential competitors	0.735		
Enterprises can effectively deal with resource use problems in a dynamic environment	0.686		
**Low-cost Innovation**		0.730	0.525
Enterprises develop new products and services at low cost	0.766		
Enterprises carry out process innovation or process innovation in a low-cost way	0.762		
Enterprises develop and utilize existing technologies and capabilities in a low-cost way	0.751		
Enterprises improve the speed of innovation with low cost	0.692		

OL, Outer Loadings; CA, Cronbach alpha; AVE, Average Variance Extracted.

**Table 2:** Means, standard deviations, and correlation coefficients.

	Mean	Standard deviation	VIF	1	2	3	4	5
Customer participation	4.064	0.543	1.621	**0.731**				
Exaptation	4.326	0.530	1.621	0.489^**^	**0.765**			
Resource flexibility	4.068	0.629	1.551	0.438^**^	0.476^**^	**0.780**		
Coordination flexibility	4.175	0.557	1.975	0.578^**^	0.561^**^	0.553^**^	**0.737**	
Low-cost innovation	4.145	0.543	-	0.573^**^	0.478^**^	0.438^**^	0.554^**^	**0.702**

N = 348, **p < 0.01; *p < 0.05 (two-tailed test). The diagonal bold data is the square root value of the corresponding AVE value of each variable.

### Descriptive statistics and correlation coefficient

According to the correlation test results (Table 2), customer participation was positively correlated with low-cost innovation, and the rationality of H1 could be preliminarily judged. A positive correlation was observed among customer participation, exaptation, and low-cost innovation. This suggests that exaptation might play a mediating role between customer participation and low-cost innovation, suggesting the rationality of hypotheses 2–4. In addition, a significant correlation was noted among strategic flexibility (resource flexibility and coordination flexibility), exaptation, and low-cost innovation, providing a basis for future empirical research. In addition, variance inflation factors (VIF) greater than 10 suggest a collinearity problem. As shown in Table 2, the VIFs between customer participation, exaptation, resource flexibility, coordination flexibility, and low-cost innovation were 1.621, 1.621, 1.551, and 1.975, respectively. The variance inflation factors (VIF) of the variables in Table 2 were much less than 10, indicating that there was no serious multicollinearity between the variables in this paper.

### Mediating effect test

A hierarchical linear regression method was used for hypothesis testing; Table 3 shows the detailed results. Specifically, model 2 examined the impact of customer participation on low-cost innovation, showing that customer participation had a significant positive impact on low-cost innovation (*β* = 0.580, *p* < 0.001), thereby verifying H1. Model 1 tested the effect of customer participation on exaptation, showing that customer participation had a significant positive impact on exaptation (*β* = 0.490, *p* < 0.001), thereby verifying H2. Model 3 tested the impact of exaptation on low-cost innovation. The regression results showed that exaptation had a significant positive impact on low-cost innovation (*β* = 0.492, *p* < 0.001), thereby verifying H3. A significant regression coefficient for customer participation in low-cost innovation was obtained in model 4 after the addition of the (*β* = 0.446, *p* < 0.001), and the absolute value of the regression coefficient was smaller than the regression coefficient of customer participation in low-cost innovation in model 2. This showed that exaptation partially mediated the effect of customer participation on low-cost innovation, thereby verifying H4.

**Table 3:** Regression analysis results.

Variable	Exaptation	Low-cost innovation			Exaptation			
	Model 1	Model 2	Model 3	Model 4	Model 5	Model 6	Model 7	Model 8
Enterprise age	0.020	0.014	−0.003	0.009	0.017	0.019	0.009	0.002
Enterprise scale	−0.068	−0.004	0.051	0.015	−0.045	−0.044	−0.039	−0.033
Ownership nature	0.074	−0.066	−0.090	−0.087	0.076	0.069	0.060	0.068
Industry distribution	0.021	−0.079	−0.079	−0.084	0.028	0.031	0.021	0.004
Customer participation	0.490^***^	0.580^***^		0.446^***^	0.347^***^	0.354^***^	0.251^***^	0.322^***^
Exaptation			0.492^***^	0.272^***^				
Resource flexibility					0.321^***^	0.288^***^		
Customer participation × resource flexibility						−0.112^*^		
Coordination flexibility							0.411^***^	0.439^***^
Customer participation × coordination flexibility								0.173^**^
Δ*R^2^*	0.250	0.340	0.246	0.396	0.333	0.344	0.361	0.382
*F*	22.754^***^	35.306^***^	22.313^***^	37.280^***^	28.313^***^	25.466^***^	32.101^***^	30.078^***^

***Significant at the 0.001 level; **significant at the 0.01 level; *significant at the 0.05 level.

We also used bootstrap sampling (bootstrap sample size = 2000) to generate the asymmetric confidence interval (CI) for indirect relationships. The results showed that the mediating effect of exaptation was 0.127, and 95% CI was [0.060, 0.216], excluding 0, suggesting that exaptation exerted a significant mediating effect. The direct effect of customer participation on low-cost innovation was 0.446, with a 95% CI of [0.351, 0.542], excluding 0, a further indication that exaptation mediated the process of customer participation in driving low-cost innovation. Hence, H4 is further verified.

### Moderating effect test

The independent and moderator variables were centrally processed when testing the moderating effect to obtain their interaction terms. First, according to models 5 and 6 in Table 3, the product term of customer participation and resource flexibility was significant and negative (*β* = −0.112, *p* < 0.05), suggesting that resource flexibility negatively moderated the correlation between customer participation and exaptation. Hence, H5a is verified. According to models 7 and 8 in Table 3, the product term of coordination flexibility and exaptation was significant and positive (*β* = 0.173, *p* < 0.01), suggesting that coordination flexibility positively moderated the correlation between customer participation and exaptation. Hence, H5b is verified. Second, the bootstrap method further tested the moderating effect of strategic flexibility, showing that the interaction coefficient of customer engagement and resource flexibility was significant (*β* = −0.170, *p* < 0.05), with a 95% CI of [−0.302, −0.037], excluding 0. When the resource flexibility was low, the indirect effect was significant (*r* = 0.453, BootLCI [0.323, 0.582]). When the resource flexibility was high, the indirect effect was significant (*r* = 0.239, BootLCI [0.118, 0.361]). Hence, H5a is further verified. Similarly, the interaction coefficient of customer participation and coordination flexibility was significant (*β* = 0.189, *p* < 0.001), 95% CI [0.081, 0.298], excluding 0. When the coordination flexibility was low, the indirect effect was significant (*r* = 0.204, BootLCI [0.102, 0.307]). When the coordination flexibility was high, the indirect effect was significant (*r* = 0.415, BootLCI [0.274, 0.556]). Hence, H5b is further verified.

### Test of the moderated mediation model

Table 4 shows the results of the moderated mediation effect test. At low resource flexibility, the mediating effect of exaptation was significant (*β* = 0.121; 95% CI [0.058, 0.212], including 0). At high resource flexibility, the mediating effect of exaptation was significant (*β* = 0.064; 95% CI [0.023, 0.137], including 0). The index value of the judgment index was −0.045, and 95% CI was [−0.103, −0.013], excluding 0, suggesting that the moderated mediation model was established. Hence, H6a is verified. Similarly, at low coordination flexibility, the mediating effect of exaptation was significant (*β* = 0.055; 95% CI [0.013, 0.126], including 0). At high coordination flexibility, the mediating effect of exaptation was significant (*β* = 0.111; 95% CI [0.059, 0.189], including 0). The index value of the judgment index was 0.050, and 95% CI was [0.010, 0.095], excluding 0, suggesting that the moderated mediation model is established and H6b is verified.

**Table 4:** Test results of the moderated mediation model.

Mediation variable	Moderator	Indirect effects under different conditions				Moderated mediation effect			
		Effect	SD	Lower limit set	Upper limit	index	SD	Lower limit set	Upper limit
Exaptation	LRF	0.121	0.038	0.058	0.212	−0.045	0.021	−0.103	−0.013
	HRF	0.064	0.028	0.023	0.137				
Exaptation	LCF	0.055	0.029	0.013	0.126	0.050	0.025	0.010	0.095
	HCF	0.111	0.032	0.059	0.189				

LRF, low resource flexibility; HRF, high resource flexibility; LCF, low coordination flexibility; HCF, high coordination flexibility.

## Conclusions, implications, and directions

### Conclusion

Based on the exaptation and strategic flexibility theories, this study constructed and verified a moderated mediation model and discussed the influence mechanism and path of customer participation driving low-cost innovation. Accordingly, the following conclusions are drawn:

First, customer participation exerted a direct and significant impact on low-cost innovation. The theoretical deduction, data test, and analysis revealed that customer participation positively impacted low-cost innovation. The research conclusion echoes and expands the view that “customer participation is an important prevariable of product innovation” put forward by Zhang and Chen (2014) and Zhang and Cai (2020). In addition, it shows the critical role played by customer participation in driving low-cost innovation. Under the open innovation mode, multi-agent interactive innovation has become a critical ecology of low-cost innovation. As a crucial source of enterprise innovation, customer participation can decrease the transaction cost and failure rate of innovation and provide reliable demand resource support for the success of product innovation and shorten the R&D cycle of new products, to promote low-cost innovation. The research conclusions refine the mechanism of the effect of customer participation on innovation and provide new ideas for the identification of paths toward low-cost innovation.

Second, exaptation mediates the correlation between customer participation and low-cost innovation. Relevant studies have discussed the mediating mechanism of customer participation affecting product innovation from the standpoint of organizational learning and knowledge-sharing (Yao et al., 2015; Zhang and Cai 2020). Few studies have investigated the internal mechanisms through which customer engagement affects low-cost innovation. Based on a new perspective, this study demonstrates that exaptation is an integral path for how customer participation affects low-cost innovation; that is, it verified that customer participation is a key predictor of exaptation (Ganzaroli and Pilotti 2011) and explained the important value of exaptation to low-cost innovation. As a crucial source of innovation, the formation of exaptation warrants maintenance, activation, and situational placement of the expansion pool, expansion activities, and expansion forum (Garud et al., 2018). In addition, customer participation can provide diversified knowledge, such as demand preference, transfer, and feedback information, and provide resources and power for establishing an expansion pool and implementing expansion activities. Meanwhile, the cooperation between customers and enterprise R&D departments to participate in innovation provides likely scenario stimulation for product function transfer. The diversified knowledge of enterprises and the search for distant opportunities in the interaction between enterprises and customers can induce function transfer (Andriani et al., 2017), resulting in product innovation behavior. In this sense, the research conclusions reveal the internal logic of customer participation driving low-cost innovation.

Third, the indirect correlation between customer participation and product innovation through exaptation will exhibit differences due to different strategic flexibility. Consistent with the findings of Li and Gao (2017), the present study introduces strategic flexibility into the correlation between “customer participation and low-cost innovation,” constructs a moderated mediation model, and further analyzes the composition path of different strategic flexibility dimensions on the impact of low-cost innovation. The test results revealed that when coordination flexibility and resource flexibility increase, the indirect effect of customer participation on low-cost innovation increases or decreases. Moreover, it should be emphasized that the previous literature took strategic flexibility as an overall variable to examine its moderation mechanism on customer participation and product innovation (Zhang and Chen 2014) and did not distinguish the differential role of different dimensions of strategic flexibility in moderating the correlation between customer participation and low-cost innovation. This study demonstrates that the correlations between coordination and resource flexibility are moderated differently. The research conclusions expand the existing theoretical framework of the role of customer participation-driven low-cost innovation and provide a new perspective for exploring the function boundary of the correlation between them.

### Theoretical implications

First, existing research on the correlation between customer participation and low-cost innovation is limited, as is the study of factors that affect the relationship. To overcome the limitations of previous studies and enable the assessment of the correlation between them from a more comprehensive standpoint, this study introduces the concept of exaptation to analyze the mechanism underlying the effect of customer participation on low-cost innovation, systematically clarifies the impact of customer participation on low-cost innovation, and demonstrates that exaptation plays a crucial bridging role between customer participation and low-cost innovation. In addition, this study builds on the research of Ren et al., (2019), and its conclusion further verifies and expands the viewpoint that “customer participation is an important pre-variable of product innovation” (Zhang and Cai 2020), and furthers the understanding of how customer participation can promote low-cost innovation. Moreover, the study addresses the problem of insufficient attention to the mediating mechanism of the correlation between the two at a macro-level and expands the application field of exaptation theory.

Second, the study reveals the moderating role of strategic flexibility (resource flexibility and coordination flexibility) in the correlation between customer participation and exaptation and demonstrates that resource flexibility and coordination flexibility can further moderate the mediating role of exaptation through different mechanisms. Then, to some extent, this study explains the problem of how to better promote customer participation and promote low-cost innovation through exaptation; this enables an understanding of the boundary conditions of customer participation and deepens the understanding of the crucial role of customer participation in the process of low-cost innovation from different perspectives. Meanwhile, the research conclusions make up for the lack of moderating mechanism of indirect correlation between customer participation and low-cost innovation and expand the application scope of strategic flexibility.

### Practical implications

First, attach importance to customer participation value and provide creative support for low-cost innovation. This would involve obtaining information on the macro-environment, industry dynamics, and market through questionnaire surveys, copywriting research, interviews, and other methods, focusing on changes in technology and customer demand and strengthening the connections between products, technology, and customer demand through various product fairs, trade fairs, entry fairs, and other forms to support product innovation. Second, establishing an interactive innovation community between enterprises and customers using mixed online/offline modes, improving communication channels and sharing mechanisms, unifying customer perception, evaluation, participation, and low-cost innovation, enhancing the likelihood of the application of existing knowledge in new fields by absorbing the knowledge of front-end consumption, and providing a knowledge-driven and situational interaction platform for low-cost innovation.

Second, drive the process of exaptation to promote low-cost innovation. First, pay attention to the important value of exaptation as the source of innovation and realize that expansion adaptation can also produce innovation, even breakthrough innovation, through the transformation of existing products (Andriani et al., 2017), assimilating traditional innovation ideas and opening up innovation paths. Second, stimulate exaptation and improve expansion adaptability through various ways. Notably, store diversified knowledge and apply existing knowledge to new situations to enhance the likelihood of exaptation. Alternatively, build an organizational structure conducive to internal and external smoothness, create a cultural atmosphere of sharing, communication, tolerance, and openness, and improve employees’ creativity and innovation.

Third, augment strategic, flexible management and provide trigger conditions for the transformation from customer participation to exaptation. If enterprises want to obtain the knowledge and power to promote exaptation from customer participation, they must break through the organizational inertia and “asset trap” that might be caused by resource flexibility and realize the agglomeration, integration, and transformation of resources in innovation; this is because it is hard to work only by occupying resources (Xu et al., 2016), so they must combine resource flexibility with the trigger conditions of expansion adaptation. In contrast, enterprises should improve coordination flexibility, augment the flexible allocation of resources and flexible allocation of processes, enhance environmental adaptability, dynamic ability, and creative problem-solving ability, and improve conditions for customer participation, and exaptation to provide resources, capabilities, and market opportunities.

### Limitations and future research

Despite its contribution to theory and practice, this study has several potential limitations. First, this study incorporated customer participation as an entire concept into the theoretical model and did not test the effect of customer participation on low-cost innovation from different dimensions (Zhang and Cai 2020). Thus, future research can further refine the research hypotheses from different perspectives and, more precisely, understand the different roles of different dimensions of customer participation in promoting exaptation and low-cost innovation. Second, as this study used cross-sectional data to study the impact of customer participation on low-cost innovation, it is difficult to examine the impact of customer participation on low-cost innovation although the time-varying process. In the future, practical sequence data can be used to explore the relationship between variables more accurately. Third, exaptation is a new research field, and the scale design of exaptation is a critical research topic; however, research on the topic is limited, indicating an urgency for further extensive investigation.

## Data availability statement

The original contributions presented in the study are included in the article/supplementary material, further inquiries can be directed to the corresponding authors.

## Author contributions

CT: construction of theoretical model and drafting the manuscript. YS: acquisition of data. XZ: analysis of data. YL: revising the manuscript critically for important intellectual content. All authors contributed to the article and approved the submitted version.

## Funding

This work was supported by Humanities and Social Science Research Youth Fund Project of Ministry of Education (19YJC630153), Hebei Social Science Fund Project (HB21GL035), Hebei Province Social Science Development Research Project (20210101001), Hebei Agricultural University Introduced Talent Research Project (YJ2021002), and Shanxi Province Education Science “Thirteenth Five-Year Plan” Project (GH-18038).

## Conflict of interest

The authors declare that the research was conducted in the absence of any commercial or financial relationships that could be construed as a potential conflict of interest.

## Publisher’s note

All claims expressed in this article are solely those of the authors and do not necessarily represent those of their affiliated organizations, or those of the publisher, the editors and the reviewers. Any product that may be evaluated in this article, or claim that may be made by its manufacturer, is not guaranteed or endorsed by the publisher.
